# Advancing Paternal Age Is Associated with Deficits in Social and Exploratory Behaviors in the Offspring: A Mouse Model

**DOI:** 10.1371/journal.pone.0008456

**Published:** 2009-12-30

**Authors:** Rebecca G. Smith, Rachel L. Kember, Jonathan Mill, Cathy Fernandes, Leonard C. Schalkwyk, Joseph D. Buxbaum, Abraham Reichenberg

**Affiliations:** 1 Medical Research Council Social Genetic and Developmental Psychiatry Centre, King's College London, London, United Kingdom; 2 Department of Psychological Medicine and Psychiatry, King's College London, London, United Kingdom; 3 Department of Psychiatry, Mount Sinai School of Medicine, New York, New York, United States of America; 4 Laboratory of Molecular Neuropsychiatry, and the Seaver Autism Center for Research and Treatment, Mount Sinai School of Medicine, New York, New York, United States of America; Chiba University Center for Forensic Mental Health, Japan

## Abstract

**Background:**

Accumulating evidence from epidemiological research has demonstrated an association between advanced paternal age and risk for several psychiatric disorders including autism, schizophrenia and early-onset bipolar disorder. In order to establish causality, this study used an animal model to investigate the effects of advanced paternal age on behavioural deficits in the offspring.

**Methods:**

C57BL/6J offspring (n = 12 per group) were bred from fathers of two different ages, 2 months (young) and 10 months (old), and mothers aged 2 months (n = 6 breeding pairs per group). Social and exploratory behaviors were examined in the offspring.

**Principal Findings:**

The offspring of older fathers were found to engage in significantly less social (p = 0.02) and exploratory (p = 0.02) behaviors than the offspring of younger fathers. There were no significant differences in measures of motor activity.

**Conclusions:**

Given the well-controlled nature of this study, this provides the strongest evidence for deleterious effects of advancing paternal age on social and exploratory behavior. De-novo chromosomal changes and/or inherited epigenetic changes are the most plausible explanatory factors.

## Introduction

Accumulating evidence from epidemiological research has demonstrated an association between advanced paternal age and risk for several psychiatric disorders including autism [1], schizophrenia [2] and early-onset bipolar disorder [3]. Despite the methodological advantages of epidemiological research, a major limitation is that techniques are limited to observation. In order to establish causality, experimental evidence in the form of randomized-controlled trials or the development of animal models is required [4]. Animal models are particularly important as they allow environmental and genetic confounds to be controlled.

The lack of complete specificity in the association between advancing paternal age and psychiatric disorders may suggest that advancing paternal age is related to phenotypes shared across disorders. One phenotype in-common to schizophrenia, autism and bipolar disorder is abnormalities in social cognition broadly defined severe social deficit [5], [6], [7], [8]. A recent epidemiological study found an association between advancing paternal age and impaired social functioning in male offspring in the general population [9].

In this study we examined the effect of older paternal age on social and non-social behavior in mice. To the best of our knowledge this is the first fully-controlled animal study of the effects of paternal age on these behaviors.

## Results

### Social Behavior

**Figure 1:**
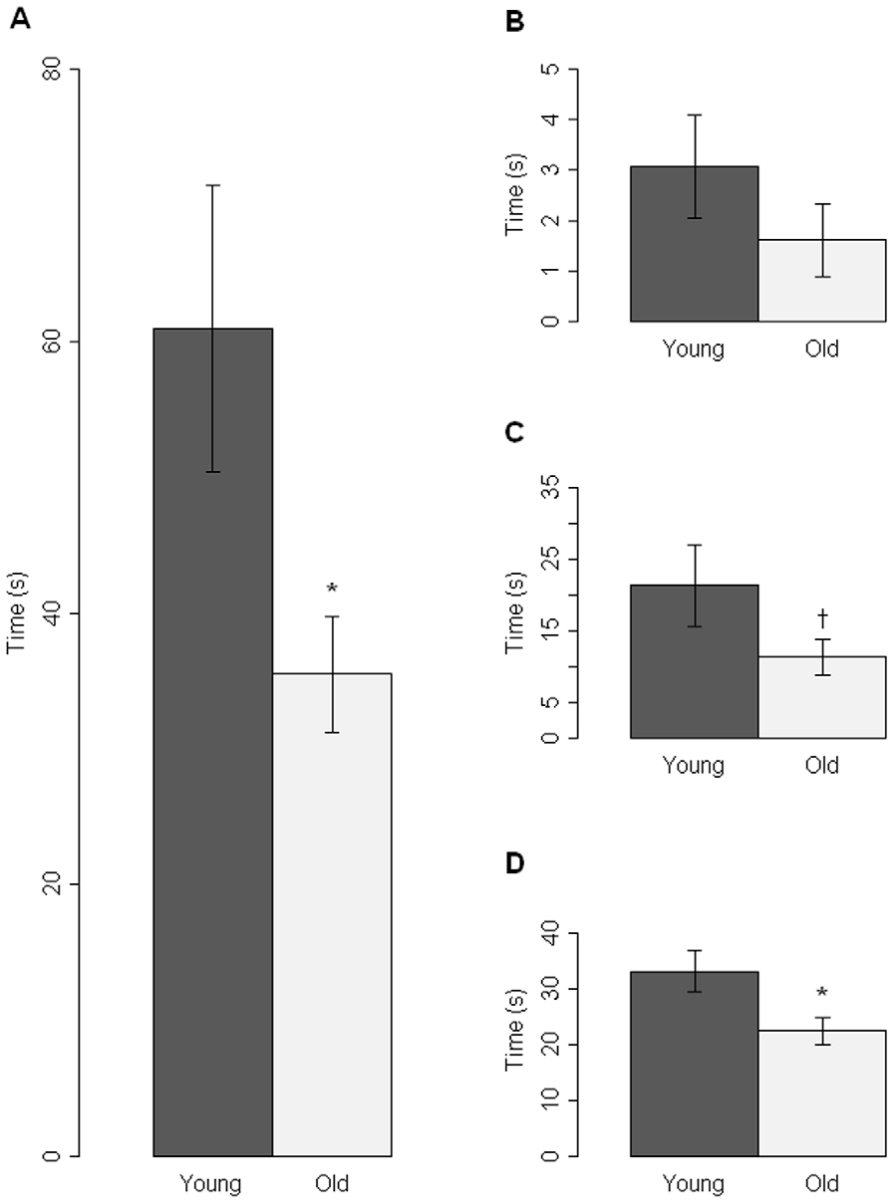
Results of social behavioral data from male offspring of young fathers (n = 12) and old fathers (n = 12). * shows a p-value of less than 0.05, † shows p-value of 0.06. A. Mean time (±SEM) displaying all social behaviors toward a conspecific mouse (broken down into components in B, C and D). B. Mean time (±SEM) displaying allogrooming behavior towards a conspecific mouse. C. Mean time (±SEM) displaying anogenital sniffing behavior towards a conspecific mouse. D. Mean time (±SEM) displaying sniffing behavior towards a conspecific mouse.

Offspring of old fathers engaged in less social activity than the offspring of young fathers, spending less time socially-interacting with the conspecific mice (t = 2.23, d.f. = 22, p = 0.02, one-tailed test, **Figure 1**). This result was consistently observed across all measures of social behavior. There were no significant differences in overall locomotor activity.

### Exploration in the Holeboard

**Figure 2:**
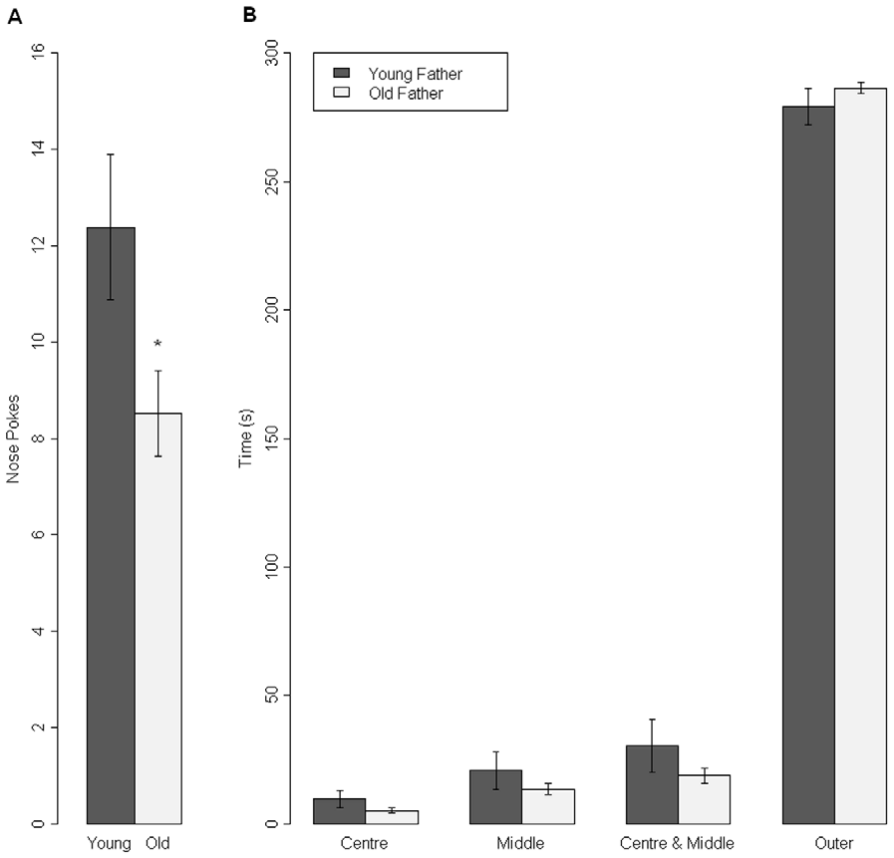
Results of holeboard and open field data from male offspring of young fathers (n = 12) and old fathers (n = 12). * shows a p-value of less than 0.05. A. Mean number of nose pokes (±SEM) into holes in the holeboard trial. B. Mean time spent in each area of arena (±SEM) in the open field task.

Offspring of old fathers demonstrated reduced exploration in the holeboard, making fewer nose pokes and spending less time nose poking than offspring of young fathers (t = −2.21, d.f. = 22, p = 0.02; **Figure 2A**). No significant differences were evident in distance moved or time spent in the centre of the Holeboard arena.

### Exploration in the Open Field

Offspring of old fathers were less exploratory in the Open Field, taking longer to enter the central zone of the arena (t = 1.7837, d.f. = 22, p = 0.04). However, there were no significant differences inthe time spent in the middle (t = −0.9548, d.f. = 22, p = 0.1785) or central zones (t = −1.3166, d.f. = 22, p = 0.1056) (Figure 2B) or in overall locomotor activity between offspring of old fathers and offspring of young fathers in the open field.

To further explore these findings we examined the same set of behaviors in a small group of mice that were the offspring of very old fathers (aged >12 months, n = 9 male offspring generated from 7 breeding pairs). The behavioral results of reduced social behavior and exploration were seen in the offspring of very old fathers, but the numbers are too small to allow for a reliable statistical test (data not shown).

## Discussion

Using a mouse model we documented deleterious effects of advancing paternal age on offspring behavior. Male offspring of older fathers engaged in less social behavior and exhibited less exploration in a novel environment. These effects were not confounded by differences in overall locomotor activity. Abnormalities in social behavior characterize psychiatric disorders previously linked to advancing paternal age, suggesting a common phenotype affected by paternal age.

There are several advantages for the mouse model used in this study. First, given the tractable nature of animal work, the environment was tightly controlled, minimizing any environmental confounds. Second, the age of all the mothers of the offspring was standard such that differences observed in the offspring cannot be accounted for by maternal age.

Finally, the most common reference inbred strain of mouse was used (C57BL/6J), reducing genetic variation.

In men, it is thought that the spermatogonial stem cell divisions occurring over the life-course of males result in higher mutational rates and cytogenetic abnormalities in the sperm of older men [10], [11]. Numerous neurological and psychiatric disorders have been related to genomic alterations [12]. A number of studies have uncovered an increased prevalence of de-novo copy-number variants (CNVs), and other forms of genomic alterations in autistic and in schizophrenia cases [13], [14].

An alternative explanation is that epigenetic dysfunction underlies some paternal age effects. Epigenetic dysfunction has been associated with several neuropsychiatric disorders, including schizophrenia and bipolar disorder [15]. A study by Flanagan and colleagues [16] reported intra- and inter-individual epigenetic variability in the male germline, and found a number of genes that demonstrated age-related DNA-methylation changes. Epigenetic signals are generally reprogrammed in the germline, although it appears that such reprogramming may not be fully complete across all regions of the genome [17]. In particular, repetitive and transposable elements in the genome, which are generally hypermethylated, are often not efficiently reprogrammed [18]. It is thus plausible that de novo structural mutations, which are often associated with repetitive DNA sequence motifs, may also be subjected to differential epigenetic reprogramming implicating both mutagenic and epigenetic processes in the phenotypic manifestation of increased paternal age.

Despite the advantages of this model, the results of this study should be interpreted in light of some limitations. We only examined one strain of male mice. This was *a-priori* decided in order to follow common practice in animal research aimed at limiting variation caused by sex differences in behaviors. Hence, findings should not be generalized across sexes. In addition, behavior was assessed at one developmental stage (12 weeks, young adulthood). Thus, the developmental nature of these differences could not be determined.

In conclusion, this study provides the strongest evidence to date for the behavioral effects of advancing paternal age on the offspring. Studies are ongoing to investigate the role of molecular changes in mediating the effects of advancing paternal age on social and exploratory behaviors in offspring, by assessing de-novo CNV events and alterations in DNA methylation.

## Methods

### Breeding Strategy

C57BL/6J mice were bred and maintained in the Biological Services Unit at the Institute of Psychiatry, Kings College London using stocks purchased from Charles River Laboratories. All housing and experimental procedures were performed in accordance with the UK Home Office Animals (Scientific Procedures) Act 1986. Typical breeding age for mice starts at 2 months. Male breeders are generally retired after 7–8 months. Therefore, females aged 2 months were bred with males of two different ages; young males of 2 months (n = 6 breeding pairs), and old males of 10 months (n = 6 breeding pairs). The average litter size within each age group was 7 (male to female ratio 1∶1) and total progeny generated was 40 mice in the young fathers group and 44 mice in the old fathers group. Two males were randomly selected from each litter (n = 12 males per group) and weaned aged 4–5 weeks and pair housed with their siblings and then individually housed for two weeks prior to testing. Mice were housed in standard cages measuring 30.5×13×11 cm, with food and water available *ad libitum*. The housing room was maintained on a standard light/dark cycle with white lights on from 08:00 to 20:00. Ambient temperature in all rooms was maintained at 21±2°C with 45% humidity.

### Offspring Behavioral Testing

Offspring were aged 12 weeks at the start of testing and all testing took place during the light phase with a light level <30 lux in the test room. Each apparatus was wiped clean with 1% Trigene® between subjects to avoid olfactory cueing behaviors. Behaviors for all tests were recorded on videotapes for further detailed analysis. Mice were returned to their home cage at the end of each test.

### Social Behavior

The social behavior of the test mice towards a juvenile conspecific was assessed in a 5 minute trial [19]. The test mouse is habituated in an arena (36×20×14 cm) for 5 minutes, after which a male juvenile conspecific of the same strain (aged 4 weeks) was introduced for a further 5 minutes. During this trial, social behavior (including social sniffing, anogenital sniffing and allogrooming) by the test mouse towards the conspecific were scored from videotape by an observer blind to the group factor of paternal age.

### Holeboard

The holeboard test is used to measure activity and exploration in a novel arena [20]. The Truscan Photo Beam Activity System (Coulbourn Instruments, Whitehall, PA) was used, which consists of an arena (25.4 cm square) and a nose poke floor with 16 holes (4×4 array) with sensor rings to track movement. The beams are spaced 1.52 cm apart providing a 0.76 cm spatial resolution. Animals were placed in the arena and the movement, the number of nose pokes and the time spent nose poking were recorded automatically by beam breaks for 5 minutes using the Truscan program.

### Open Field

The open field [21] used a square white acrylic box with dimensions 72×72×33 cm. The animal was placed in the outer part of the arena facing an outer wall and allowed to freely explore the arena for 5 minutes. A video camera placed above the arena allowed movement to be tracked using an automated tracking system (Ethovision, Noldus Information Technologies). The number of faecal boli and urination were recorded at the end of the test. A square of equal distance from the periphery (36×36 cm) was defined in Ethovision as the ‘outer’, ‘middle’ and ‘central’ zones in order to determine the number of entries into, and time spent in, these zones in the arena. In addition, the latency to enter the inner zones as well as locomotor activity in all three zones of the arena were measured by the tracking system.

### Statistical Analysis

Behavioral performances of offspring of young fathers and offspring of old fathers in the social interaction task, holeboard and open field were compared using unpaired, one-tailed Students t-tests. Significance level was set at 0.05.
